# Monthly Periscope

**Published:** 1854-01

**Authors:** 


*Monthly Periscope.*—*Fistula Lachrymalis.*—We notice in a letter from Paris, appearing in the Boston Medical and Surgical Journal, an account of a new operation for this surgical affection. The unsatisfactory results attained by the style, have previously suggested the trial of other modes of cure. Among these, the introduction of a small sound, and catheterism, appeared most likely to succeed. The dilatation of a bony canal is, however, no easy matter. In a conversation with Prof. Hamilton, a few days since, relative to the dilatation of the Eustachian tube, he assigned as reasons for its failure, the bony nature of a portion of that passage, and the consequent fact, that a sound could only press the mucous membrane against the bone for the moment, leaving it to resume its swollen condition as soon as the instrument was removed, with the added irritation due to the presence of a foreign body.

These remarks will apply with equal force to fistula lachrymalis, and furnish a sufficient reason for the failure of the usual and authoritative means of cure.

An operation is now practised by M. Desmarres, of which we furnish our readers a brief account, leaving them to judge of the propriety of its adoption.

He "makes a somewhat circular incision down upon the sac, dissecting laterally, so that it may be fully exposed, then applies the actual cautery, which completely destroys the sac—the wound healing without any very noticeable deformity—and in twenty to forty days the patient is cured of an affection that may have lasted him many years."

The question now arises as to what becomes of the tears. Of this the letter-writer in question argues, that the ordinary evaporation from the conjunctival surface will be sufficient to keep the cheek dry under ordinary circumstances, but that when the secretion of the lachrymal gland is increased by the action of the passions, or by the presence of an irritation in the eye, it will, of course, flow over upon the cheek. This, however, occurs under the healthy condition. The propriety of the operation can be settled, by deciding as to whether it is more conducive to comfort to do without a nasal duct, or to suffer the inconveniences of fistula.

*Salines in Dysentery.*—The Ohio Medical and Surgical Journal attributes the causation of many cases of dysentery and serous diarrhœa, to torpidity of the liver, owing to deficiency of the alkalies, as compared to the fatty acids for the production of bile. This is a practical development of Bernard's physiological discovery. It is only necessary, first, to prove that a torpid liver is a cause of dysentery, This we believe ourselves, but other wiser men do not. We suppose, however, that all observers are agreed, that due attention to the chemical wants of the blood is a greatly neglected therapeutical necessity. It would seem from some casual investigation of this matter, that there was a deficiency of the salines in all that class of zymotics represented by dysentery and intermittents.

It should be recollected that the Ohio Journal advocates, not the saline cathartic, but the use of small doses of the neutral salts with a view to their absorption. If this theory be true, it is, perhaps, as well to the chemical, as to the cathartic action of this remedy, that the well known efficacy of the saline cathartic may be attributed. Of one thing we are assured, by a somewhat extended observation of many cases of dysentery in different localities—the Rochelle and Epsom salts are two of the most valuable of our remedies in that disease.

We submit that the following prescription, spoken of by the Ohio Journal as most efficient in dysentery, is hardly capable of accurate estimation of its results:

℞ Chloride of sodium, ʒss.

Sulph. morph., gr. $\frac{1}{3}$.

Repeated every four to six hours.

Those who advocate the pure opiate treatment of dysentery would ascribe greater efficacy to the one-third grain of morphia, than to a quantity of common salt, less than most people consume with their daily dinner.

*Expectant Treatment of Rheumatism.*—Can we jugulate an acute rheumatism? Those who are settled in the belief of the self-limitation of disease will readily answer no. But it is by no means proved that even self-limited diseases must necessarily occupy a certain period of time, without reference to the remedies employed. There is, or should be, a distinction between a natural and artificial limitation of disease. If we had any faith in logical medicine, we should argue that, if by the officious and improper use of medicinal agents we are able to kill our patients, thus breaking in upon the natural process of cure, we are also able, by their skilful and judicious employment, to hasten the progress of development, elimination, and cure.

These ideas are suggested by the perusal, in the Virginia Medical and Surgical Journal, of a translation from the French of J. Delwalsche, of a paper on the expectant treatment of acute articular rheumatism, pursued at the Military Hospital of Antwerp.

A reference to the opinions of writers, and to the pages of journals, will show a remarkable difference of opinion as to the proper treatment for this affection. In this connection, M. Delwalsche quotes from his predecessor in this movement—M. Gouzee—as follows:

"Of late years, there have been recommended the most violent and disproportionate modes of treating acute articular rheumatism. Tartarized antimony, nitrate of potash, blood-letting, opium, iodide of potassium and sulphate of quinine, have been employed in frightfully enormous doses; and, surely, it cannot be asserted, that a large proportion of the patients subjected to these various modes of treatment have been cured *tuto, citoque, jucunde.* It is even said that sulphate of quiaia, administered in Rasorian doses, has occasionally jugulated the patient instead of the disease.

"I have long employed a simple expectant treatment in this disease, and a year has never elapsed without my having cause for astonishment at the facility and promptitude with which my patients recovered."—(*Archives Belges*, Jan. 1844, p. 7.)

Our own experience would lead to a somewhat different conclusion; but, as he who has only witnessed disease under the operation of medicine, knows little of its real tendencies, we waive any objections on that score, and will give to this new idea a place in the record, as furnishing, at any rate, some information as to what may be the tendencies of rheumatism when left to hygiene and nature.

The clinical notes of six illustrative cases are given, all of them resulting in a cure in from eight to fifteen days after the attack. The treatment was purely expectant, consisting of diluent drinks, taken cold, or warm, as the patient preferred, with fomentations, or baths to the affected parts. The whole array of active medicinal agents is ignored, and the disease left to the curative operations of nature, aided by diluents, diet, and the recumbent posture.

It is claimed that this has been an eminently successful management—that even in those cases where cardiac symptoms supervened, it was sufficient for every emergency.

Some years ago we had some experience in the antimonial treatment. It is proper to mention here, that whenever we have employed the tartar emetic in an acute inflammation, we have limited its use to a few—usually six—hours. During this period, just enough of tartar-emetic was given to keep the patient constantly and thoroughly nauseated. On coming out of this extremely disagreeable treatment, we have repeatedly witnessed a subsidence of the articular swelling and pain, followed by convalescence in two or three days. Warm fomentations were diligently applied as adjuvants, and usually a large Dover's powder was given at bedtime.

The conclusion which we derive from these apparently opposite experiences is: that the treatment in either case rests on a similar *rationale.* M. Gouzee regards rheumatism as a blood poison, to be eliminated by unaided nature. The antimonial treatment is supposed to fill the several indications of depressing the hearts action, and eliminating the poison by increased secretion from the skin and mucous membranes.

We subjoin the conclusions arrived at by the writer under notice:

"1. Acute articular rheumatism has a natural tendency to terminate in the course of one or two weeks.

"2. Treated on an expectant plan by simple hygienic and expectant precautions, it pursues its march without danger, and ceases as soon, if not sooner, than when combatted by active measures.

"3. It is not proven that the active treatments recommended in this disease are useful, or even innocent.

"4. The cardiac murmurs which are frequently observed in the course of rheumatism, disappear spontaneously in the great majority of cases, in proportion as the disease ameliorates, and under the influence of the simplest treatment.

"5. It is far from being demonstrated that these sounds are always the signs of endocarditis."

*The Physiology of Gaseous Absorption.*—It is daily becoming more evident, that to a clear and rational physiology must we look for further advances in medical art. With this conviction strong within us, we watch with a lively interest those developments which are, from time to time, making in the chemistry of the human system. Foremost among those devoted to this section of inquiry, we recognize the name of M. Claude Bernard, of the College of France.

It is principally to the reports of American physicians, residing in Paris, that we must look for information regarding these researches. Two years since, this Journal contained a series of letters addressed to it by Prof. Jno. C. Dalton, then in Europe, detailing the very interesting experiments of M. Bernard, on the physiology of digestion. These experiments and vivisections have since been reproduced in this country, with such additions and variations as were suggested by increased experience; first, at the Buffalo Medical College, and subsequently, (during the present winter,) at the College of Physicians and Surgeons in New York, by Prof. Dalton, of the Buffalo Medical school.

M. Bernard is now engaged—as we see by published letters from Paris—upon the subject which constitutes the heading of this sketch. We find in the Boston Medical and Surgical Journal, an account of sundry experiments furnished to it by a Paris letter-writer, and of which we furnish a brief *resume*, as is the custom of our Periscope department. Oxygen is a large constituent in the organization, it being necessary to it, not only as a nutrient, but to support that combustion which maintains the vital heat. Its absorption is universal, both in the animal and vegetable world; without it development cannot progress, or germination be accomplished. The spawn of a frog, or the egg of a bird, deprived of contact with oxygen, no longer germinates, and experiment proves that, during the germination of the ova we have mentioned, oxygen is absorbed by them.

A mode of ingress for this gas is provided in the lung, where an immensely large area of absorbing surface is presented to its contact. It is a chemical property of many liquids to absorb gases presented to them. Water possesses this quality. 1000 volumes of water will, when agitated in the air, absorb 9¼ volumes of oxygen. But the absorbent capacity of different liquids varying, it is found that the same quantity of blood, thus agitated, will take up 130 volumes of oxygen.

In stating that so much blood will absorb so much oxygen, we by no means occupy the whole ground. It is not probable that all the constituents of the blood absorb a like proportion. The theory of Liebig assigned to the blood globules alone this affinity. Others have ascribed to the iron of the globules a part of the *role*. Again is this addition of oxygen to blood, a mere solution of the gas, as happens in sundry other instances, or is it properly a chemical combination, modified by the condition and constitution of the absorbing fluid? These changes of the constituents of the blood, are constantly occurring. It is found, that in the presence of certain elements, more oxygen is taken up than in their absence. This is illustrated by the chemical fact, that some salts of iron impart to water a greater capacity for gaseous absorption than it naturally possesses. It is argued, that if this absorption is a mere dissolution of the gas, like that of carbonic acid gas in water, compression would increase the amount taken up, as it does in the instance of the carbonic acid. But it is found that compression exerts no appreciable influence upon the absorption of oxygen by blood.

It was estimated by Lavoisier and Seguin, that during twenty-four hours, 20,000 grains of oxygen might enter the blood of the healthy adult. This quantity—sufficient in itself for all the purposes of life—is, however, found to vary very considerably. The conditions of digestion, of abstinence, of different qualities of food, and as it now occurs to us, of pregnancy and lactation, exert a material influence on this process. Thus, during abstinence, there is a more rapid absorption of oxygen, with a less copious evolution of carbonic acid gas than during digestion. In pregnancy and lactation, (we write from recollection of some experiments made in France some years ago, relative to the vicarity of the functions of menstruation and respiration,) the proportion is reversed, more carbonic acid being exhaled during pregnancy, than during menstruation.

Before detailing the influence of different articles of diet upon gaseous absorption, let us inquire what effect the presence of these articles has upon the absorption of gas by blood withdrawn from the system.

M. Bernard fills two tubes, or syphons, with an equal quantity of blood, drawn from the jugular vein of a dog, a syringe being used to prevent atmospheric contact. To one of these tubes a small quantity of undissolved grape sugar is added. The blood in this tube absorbed twelve divisions of oxygen—by the tube not sugared fourteen divisions were absorbed. The sugar having subsided, on a renewed agitation, eight divisions were absorbed in the sugared blood, and eleven divisions by the other.

Other *savans* have placed this experiment in a different and more satisfactory shape, but confirmatory of Bernard's experiment. It is found that rabbits, living on their natural food, which contains much sugar, for every 100 parts of oxygen absorbed, expell 91.9 of carbonic acid gas; leaving only 8.1 to be assimilated. While fasting, on the other hand, 31 parts were assimilated, and 59 evolved in carbonic acid.

It is thus proved that the presence of sugar in the blood gives rise to a marked decrease of absorption of oxygen.

It is supposed (though no experiments have been directed to this point especially) that the presence of saline ingredients in the blood will increase gaseous absorption. This is *analogically* proved by certain experiments, instituted by the French Minister of War, with reference to the effect of salting of forage for the use of horses. The result of this experimentation brought out the very rational conclusion, that salt stimulated the appetite, and induced the cattle to consume bad forage, but that it did not add to the nutritive qualities of the food.

M. Bernard has nevertheless tested the effect of chloride of sodium on gaseous absorption out of the body. The result is, briefly, that salt augments the absorption of oxygen, in the proportion of 32 parts of 100 absorbed by saline blood, to 20 parts of 100 absorbed by pure blood.

We have thus—as briefly as the importance of the subject permitted—given a *resume* of those facts in this connection which are already proved by experiment. It is uot difficult to see that these facts, with others, linked in the same chain but yet to be drawn forth, must have an important therapeutical bearing. Thus, in diabetes, a disease in which the blood is surcharged with sugar, and in which it is proved that there is a deficient absorption of the "air vital," we should look—secondarily to the primary lesion in the nervous system—to the introduction of such elements into the blood, as will quicken the absorption of oxygen, and decompose the free sugar in the circulation. It is believed that the first indication may be attained by the use of salines. The second we have, in some previous number, mentioned as already attained and proven, in the use of rennet, which converts the free sugar into lactic acid. M. Bernard supposes that the use of salt in increasing the absorbent properties of the blood, does also increase the rapidity of combustion, and consequently of emaciation.

We have already extended this sketch to a greater length than we anticipated. But before leaving it, it is proper to mention, as bearing on the causation of epidemics, the modifications which the oxygen of the atmosphere may undergo from meteoric causes. During the prevalence of cholera, a few years ago, its origin was ascribed to the presence of ozone, which is oxygen electricized. Its properties are those of an irritant gaseous acid; its respiration producing bronchitis. It has been found, on subsequent investigation, that ozone, so far from having a tendency to the excitation or development of putrefaction, or marsh miasms, is actually an antiseptic.

*The Sympathetic Nerve and its Correlate Functions.*—In addition to the theory of gaseous absorption, of M. Bernard, we find also in the St. Louis Medical and Surgical Journal, a letter from Paris, detailing his recent inquiries into the functions of the great sympathetic nerve. We therefore (in continuation of our last month's essay on Nervous Sympathy) will, in this sketch, take up the subject of the ganglionic system in its relations to reflex action.

The theory of Marshall Hall, now generally received, assigns to this claim of ganglia a function dissimilar to that of the brain or spinal cord. It is in part a motor nerve, governing the muscular actions of the thoracic and abdominal viscera. It has also a sensory function, analogous to cerebral sensibility, but capable of only one expression—that of want, as hunger, *besoin de respirer*, or the disposition to evacuate an excretory reservoir. It is probable, or perhaps it is proved, that all sensations of pain, referable to these viscera, are the results of cerebro-spinal action. We are aware that this is not essentially the theory of Marshall Hall, but will allow the discussion of this argument to merge into the history of Bernard's experiments. Dr. Hall insists upon a filamentous connection, a nerve of reflection, existing between the anterior and posterior spinal roots; not because such a nerve has ever been demonstrated, but because it was necessary to prove some connection.

Those who have witnessed the experiments of Marshall Hall upon the frog, will recollect that the pinch of the forceps, though applied to a limited space, was able to put in action the muscles of an entire limb. M. Bernard urges that so limited an irritation could not act on all the motor nerves of a limb, and argues thence that the movement is the result of a sensation.

Where does this sensation reside? Not in the brain, for the communication is cut off. To find, then, some answer to this question, is important.

If the spinal cord of a frog be cut, it loses volitional control of the muscles. They will, however, contract on an irritation applied to the foot. Destroy the sensory ganglion of the posterior root, and you destroy all motion. Irritate them, and you increase the motion. Here, then, resides this sensation. Strychnia acts upon these intervertebral ganglia, and tetanus is developed within them.

The cutting of the spinal cord involves the actual destruction of the *nodus vitæ*, which is assuredly a fault in any experiments directed to the sympathetic nerve, inasmuch as it only isolates the brain, leaving the intimate connection between the spinal and ganglionic systems unimpaired. It is desirable to avoid this—to destroy the action of the brain and cord, leaving the ganglia of the sympathetic susceptible to impressions. M. Bernard tells us that it had been hoped to find in chloroform such an agent. This poison, however, does not meet the indication. It overwhelms alike all nervous functions. In people who have been hanged, a ciliary motion of the epithelium of the trachea has been observed for thirty-six, or even forty-eight, hours after death. But in animals dying from chloroform, this motion stops immediately. Its primary and dangerous effect is undoubtedly produced upon the ganglionic system. Hence the failure of artificial respiration to recover those dying from it. There is an instantaneous paralysis of the nerves of heart and lung motion, which cuts off hope of recovery in proportion to its completeness.

The writer whose monograph forms our text, says, that M. Bernard uses "*curare*" as a toxic. What "curare" is we do not know, but as it is precisely similar in its effects to *woorari,* we fancy that *curare* is a typographical fiction, an idea confirmed by sundry other translators and printers' errors, among which we find the grey fibres of the sympathetic, mentioned as the "fibres of remark," without any regard to the memory of the illustrious Remak.

There is such a minute interweaving of the ganglionic with the cerebro-spinal system, (for the posterior ganglia of the spinal cord belong to the former, and are uniformly mentioned by Bernard as such, without specification,) that it seems well nigh impossible to isolate it. This M. Bernard has done with the woorari. Under the operation of this poison, the cerebro-spinal axis is deprived of any functions, while, the sympathetic still acting, we have organic life going on. This condition of things opens the way to new and varied experiment. Of course respiration is stopped, depending, as it does, on cerebro-spinal action; but the chain of chemical and physiological changes, which constitute the objects of respiration, remain excitable by artificial respiration.

Thus M. Bernard is able to appreciate what portion of the chemical changes in the fluids is due to the ganglionic system. He can voluntarily diminish or increase the lachrymal or urinary secretions. He can so exaggerate the secretion of sugar in the liver, as to cause diabetes to be manifested in one hour. We quote one experiment with the woorari, which, though it lacks the test of chemical experiment which is applied in some other cases, goes to show, that while the motion of muscles of organic life may be destroyed by the poison, the germ of their reproduction, and the power of governing and changing those secretions which are the final cause of that motion, is due to the sympathetic nerve.

"*Experiment.*—A dog, of middle size, poisoned before the lesson with curare, was on the point of breathing his last. M. Bernard placed him upon the table, opened his trachea, and adapted to it a tube communicating with a bellows. The blood had become black, and respiration had ceased; then insufflations were commenced in a manner to imitate and replace the movements of respiration. The blood became again sensibly red, the eyes, which were dull, reappeared, animated, and sensible to sight. The insufflations were suspended, the phenomena of death manifested themselves anew, the blood reassumed its dark color. The manometer of M. Majendie was applied, and it was proved by the elevation of the mercury, that in proportion as the insufflations were resumed and continued, the pulsations of the heart returned, and acquired a greater intensity; the heat persisted, the muscular contractions manifested themselves, the phenomena which pertain to the secretions were seen to return."

Some practical deductions remain to be considered, which we shall postpone to a future number. S. B. H.

